# 

**Published:** 1893-09

**Authors:** 


					﻿THE
Southern Medical Record
A Monthly Journal of Medicine and Surgery.
VOL. XXIII.	Atlanta, Ga., September, 1893.	NO 9.
EDITORS:
A. W. GRIGGS, M. D.
j. mcfadden gastón, m. d.
WILLIS F. WESTMORELAND, M. D.
LOUIS H. JONES, M. D.
DAN. H. HOWELL, M. D., Bus. Mgr.
TERMS : $2.00 Per Annum ; Single Copy 20 Cents.	Contents on Page Ö.
Entered at the Postoffice at Atlanta, Ga., as Second-Class Matter.
Mutual Printing Company, Publishers, 27 East Hunter St., Atlanta, Ga.
				

